# Supplementary material for: Predicting visual function by interpreting a neuronal wiring diagram
Source: Nature. 2024 Oct 2;634(8032):113–23. doi: 10.1038/s41586-024-07953-5 (PMC11446822; doi:10.1038/s41586-024-07953-5)
Supplement: Supplementary file 6 — CRF and ERF predictions for individual TmY4 and TmY9 cells. Analogous to Supplementary Data 3, but for TmY target types. Shown are the top four monosynaptic pathways, the strongest pathway passing through each of the top ten intermediary types (ranking from Extended Data Fig. 7), and the trisynaptic pathway Tm1–TmY–Dm3–TmY (see the section entitled Prediction of spatial normalization). [file 41586_2024_7953_MOESM6_ESM.zip › DataS4/TmY9qΓèÑ/720575940627681850.pdf]

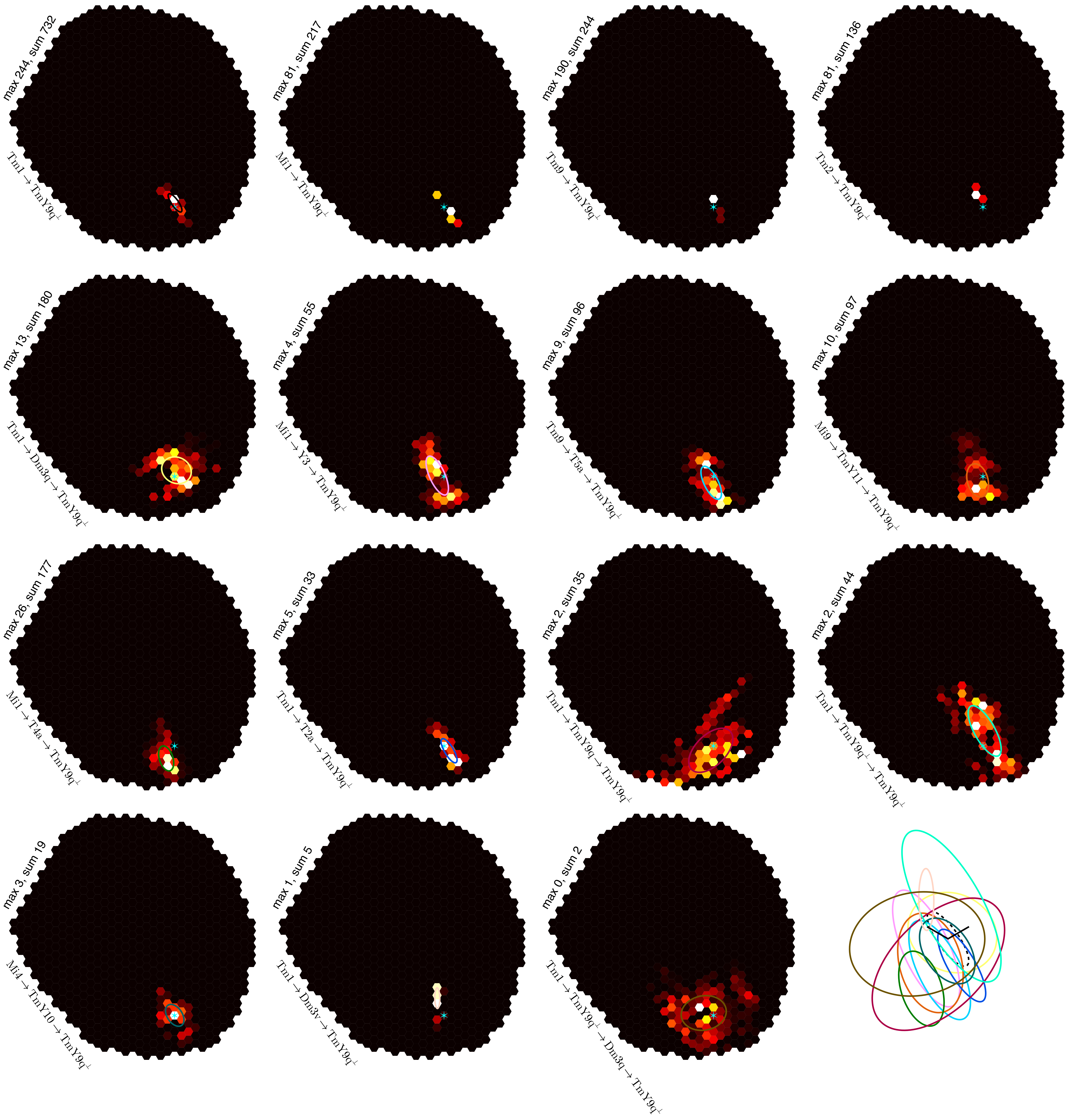

max 244, sum 732
Tm1 → TmY9q⊥
max 81, sum 217
Mi1 → TmY9q⊥
max 190, sum 244
Tm9 → TmY9q⊥
max 81, sum 136
Tm2 → TmY9q⊥
max 13, sum 180
Tm1 → Dm3q → TmY9q⊥
max 4, sum 55
Mi1 → Y3 → TmY9q⊥
max 9, sum 96
Tm9 → T5a → TmY9q⊥
max 10, sum 97
Mi9 → TmY11 → TmY9q⊥
max 26, sum 177
Mi1 → T4a → TmY9q⊥
max 5, sum 33
Tm1 → T2a → TmY9q⊥
max 2, sum 35
Tm1 → TmY9q → TmY9q⊥
max 2, sum 44
Tm1 → TmY9q⊥ → TmY9q⊥
max 3, sum 19
Mi4 → TmY10 → TmY9q⊥
max 1, sum 5
Tm1 → Dm3v → TmY9q⊥
max 0, sum 2
Tm1 → TmY9q⊥ → Dm3q → TmY9q⊥
